# Nicotine-Mediated Recruitment of GABAergic Neurons to a Dopaminergic Phenotype Attenuates Motor Deficits in an Alpha-Synuclein Parkinson’s Model

**DOI:** 10.3390/ijms24044204

**Published:** 2023-02-20

**Authors:** Jessica IChi Lai, Alessandra Porcu, Benedetto Romoli, Maria Keisler, Fredric P. Manfredsson, Susan B. Powell, Davide Dulcis

**Affiliations:** 1 Department of Psychiatry, University of California San Diego, La Jolla, CA 92093, USA; 2 Department of Drug Discovery and Biomedical Sciences, University of South Carolina, Columbia, SC 29208, USA; 3 Department of Neurobiology, Barrow Neurological Institute, Phoenix, AZ 85013, USA

**Keywords:** nicotine, dopamine, tyrosine-hydroxylase, alpha-synuclein, neurotransmitter-switching, substantia nigra

## Abstract

Previous work revealed an inverse correlation between tobacco smoking and Parkinson’s disease (PD) that is associated with nicotine-induced neuroprotection of dopaminergic (DA) neurons against nigrostriatal damage in PD primates and rodent models. Nicotine, a neuroactive component of tobacco, can directly alter the activity of midbrain DA neurons and induce non-DA neurons in the substantia nigra (SN) to acquire a DA phenotype. Here, we investigated the recruitment mechanism of nigrostriatal GABAergic neurons to express DA phenotypes, such as transcription factor Nurr1 and DA-synthesizing enzyme tyrosine hydroxylase (TH), and the concomitant effects on motor function. Wild-type and α-syn-overexpressing (PD) mice treated with chronic nicotine were assessed by behavioral pattern monitor (BPM) and immunohistochemistry/in situ hybridization to measure behavior and the translational/transcriptional regulation of neurotransmitter phenotype following selective Nurr1 overexpression or DREADD-mediated chemogenetic activation. We found that nicotine treatment led to a transcriptional TH and translational Nurr1 upregulation within a pool of SN GABAergic neurons in wild-type animals. In PD mice, nicotine increased Nurr1 expression, reduced the number of α-syn-expressing neurons, and simultaneously rescued motor deficits. Hyperactivation of GABA neurons alone was sufficient to elicit de novo translational upregulation of Nurr1. Retrograde labeling revealed that a fraction of these GABAergic neurons projects to the dorsal striatum. Finally, concomitant depolarization and Nurr1 overexpression within GABA neurons were sufficient to mimic nicotine-mediated dopamine plasticity. Revealing the mechanism of nicotine-induced DA plasticity protecting SN neurons against nigrostriatal damage could contribute to developing new strategies for neurotransmitter replacement in PD.

## 1. Significance Statement

Tobacco smoking and Parkinson’s disease (PD) have been associated with a mechanism of nicotine-induced neuroprotection of dopaminergic (DA) neurons against nigrostriatal (SN) damage. This study revealed that nicotine exposure led to a transcriptional TH and translational Nurr1 upregulation within a pool of SN GABAergic neurons in wild-type animals. In PD mice, nicotine treatment increased Nurr1 expression, reduced the number of α-syn-expressing neurons, and simultaneously rescued motor deficits. We successfully induced DA expression within SN GABA neurons in the absence of nicotine exposure by prolonged depolarization and concomitant Nurr1 overexpression. Revealing the recruitment mechanism of nigrostriatal GABAergic neurons to express DA phenotypes and the concomitant effects on motor function could contribute to developing new strategies for neurotransmitter replacement in PD.

## 2. Introduction

Parkinson’s disease (PD) is characterized by a progressive neurodegeneration of dopaminergic (DAergic) neurons [1,2,3] and aggregation of α-synuclein in the substantia nigra (SN) [4,5], comprised of the SNc (pars compacta) and the SNr (pars reticulata). Parkinsonism, the collective term for PD motor deficits including bradykinesia, tremor, rigidity, and postural instability, is a consequence of an impaired nigrostriatal pathway [6,7] due to a degeneration of SNc DAergic projection to the dorsal striatum [1,8]. Conventional and novel pharmacological treatments providing PD symptomatic relief have been developed [9,10,11,12]; however, no disease-modifying strategies exist. Neuroprotection targeting classes of compromised neurons in PD and alleviating debilitating movement disorders have been previously investigated [13,14]. Extensive evidence supports an inverse correlation between PD and cigarette smoking [15,16,17,18] and that nicotine mediates neuroprotection when administered before or during nigrostriatal damage both in rodents [19] and primates [20].

Nicotine activates nicotinic acetylcholine receptors (nAChRs) and regulates the function of neurons by increasing calcium influx and inducing neuronal depolarization [21,22]. Nicotine-mediated calcium signaling occurs via direct calcium influx through nAChRs, indirect calcium influx through voltage-dependent calcium channels, and intracellular calcium release from internal stores [23,24,25]. A number of nAChR subtypes are expressed in both DAergic and GABAergic neurons in the SNc and SNr [26,27,28] and nicotine-induced neuroprotection can be mediated by heteromeric α4* (primarily α4β2*) and homomeric α7 receptors [29,30]. Importantly, chronic nicotine exposure upregulates α4* nAChRs localized in SNr GABAergic neurons without changing the α4* nAChRs levels in SNc DAergic neurons [28], suggesting that nicotine might initiate selective activity-dependent signaling on SNc and SNr neurons during chronic exposure. 

The expression of the transcription factor Nurr1 (NR4A2), which is essential for the acquisition [31] and maintenance [32] of the DAergic phenotype, might participate in the mechanism of nicotine-mediated neuroprotection of nigrostriatal neurons. Studies have shown that Nurr1 expression is regulated by calcium-mediated neuronal activity [33] and increases in the striatum in response to chronic nicotine administration [34]. Importantly, Nurr1 plays a significant role in neuronal survival [35] and NR4A-deficient neurons are generally more sensitive to neurodegeneration due to the downregulation of NR4A-dependent neuroprotective gene programs [36]. Emerging evidence indicates that impaired Nurr1 expression might contribute to the pathogenesis of PD [37,38]. Due to its neuroprotective role for DAergic neurons, Nurr1 has been identified as a therapeutic target for PD. Remarkably, it was found that Nurr1 agonists improve behavioral deficits in a PD rat model [39]. Preclinical studies have also shown a promising role of Nurr1 in next-generation PD treatments, including Nurr1-activating compounds and Nurr1 gene therapy aimed at enhancing DA neurotransmission and protecting DAergic neurons from cell damage by environmental toxins and neuroinflammation [37,40,41]. 

Here, we investigate the cell-specific mechanism through which chronic nicotine influences the activity-dependent regulation of genes controlling the expression of Nurr1 and the DA-synthesizing enzyme tyrosine hydroxylase (TH) in neurons of the SN, while attenuating some of the PD-associated locomotor deficits. We then tested whether artificially recreating the cellular environment that mimics nicotine-mediated exposure in untreated wild-type mice is sufficient to induce dopamine plasticity in the SN. 

## 3. Results

### 3.1. Chronic Nicotine Exposure Attenuates PD-Associated Locomotor Deficits and Increases Nurr1 Expression in the SNr

**Figure 1:**
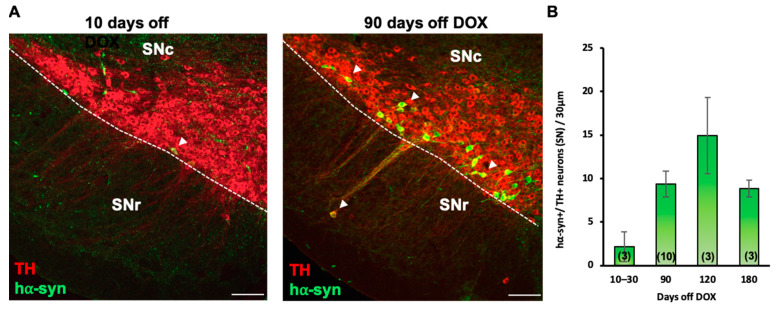
Inducible human A53T alpha synuclein accumulates over time. (**A**) Confocal images of coronal section (30 μm) through the substantia nigra compacta (SNc) and reticulata (SNr) of the *PITX3-IRES2-tTA/tetO-A53T* double-transgenic mice showing a substantial increase of hα-syn expression after 90 days off doxycycline (DOX) when compared to 10 days off DOX. Colocalization shows that hα-syn was selectively expressed in the TH+ cells (arrowheads) in the SN. Scale bars = 100 μm. (**B**) Quantification of neurons displaying hα-syn /TH colocalization shows a 2-fold increase in hα-syn expression after 90 days off DOX and a 3-fold increase after 120 days. The expression after 180 days off DOX was comparable to 90 days off DOX. Graphs show mean ± SEM. The number of animals is annotated in parentheses for each condition.

**Figure 2:**
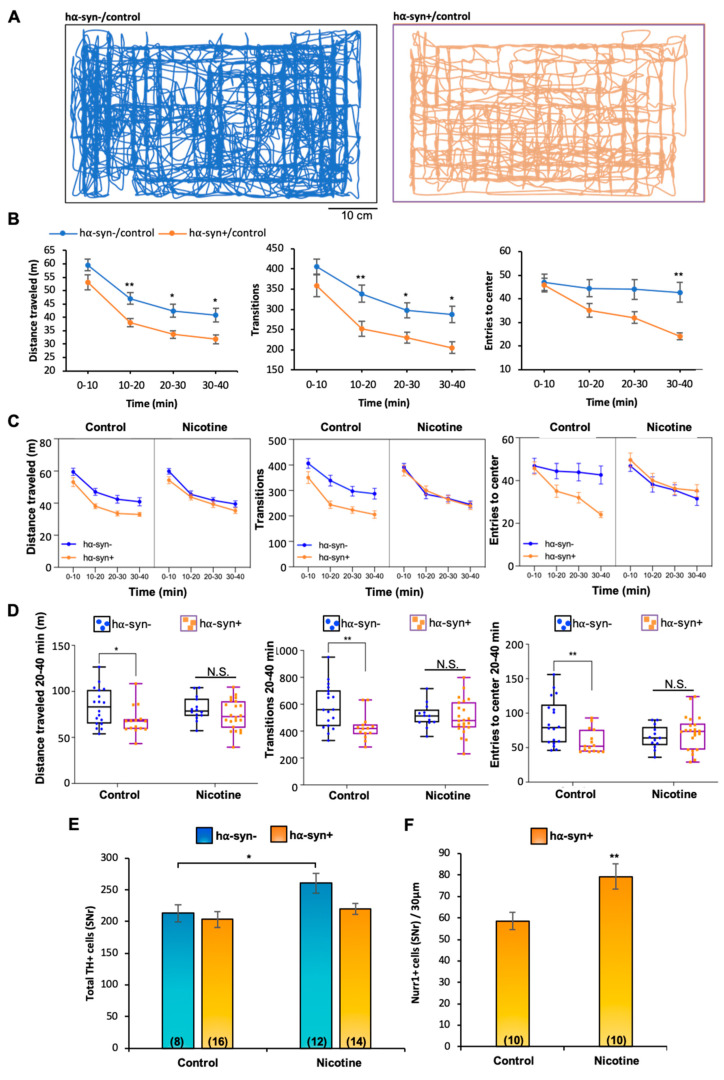
Chronic nicotine exposure attenuates locomotor deficits and increases SNr Nurr1 expression in Pitx3-A53T transgenic mice. (**A**) Spatial patterns of locomotion analyzed with the Behavior Pattern Monitor (BPM) within 10 min duration of the testing session exhibited by nicotine-untreated (control) Pitx3-A53T transgenic (hα-syn+, **right**) and a hα-syn- (**left**) mice. (**B**) Locomotion measures from 0 to 40 min of the BPM testing session show that hα-syn+/control mice displayed significant locomotor deficits, including distance traveled (two-way ANOVA, main effect of hα-syn: F_(1,30)_ = 8.463, *p* < 0.01, Bonferroni’s Multiple Comparisons: hα-syn-/control vs hα-syn+/control at 10–20 min: *p* < 0.01, 20–30 min: *p* < 0.05, 30–40: *p* < 0.05), number of transitions across different regions of the chamber (F, two-way ANOVA, main effect of hα-syn: F_(1,31)_ = 8.143, *p* < 0.01, Bonferroni’s Multiple Comparisons: hα-syn-/control vs hα-syn+/control at 10–20 min: *p* < 0.01, 20–30 min: *p* < 0.05, 30–40 min: *p* < 0.05), and entries to the center of the chamber (two-way ANOVA, time x hα-syn interaction: F_(3,91)_ = 3.266, *p* < 0.05, main effect of hα-syn: F_(1,32)_ = 6.114, *p* < 0.05, Bonferroni’s Multiple Comparisons: hα-syn-/control vs hα-syn+/control at 30–40 min: *p* < 0.01). Every measure shows a main effect of time, *p* < 0.0001. Graphs show mean ± SEM: * *p* < 0.05, ** *p* < 0.01. (**C**,**D**) Chronic nicotine exposure attenuated locomotor deficits as no significant differences in these measures were observed between nicotine-exposed hα-syn- and hα-syn+ groups. (**C**) Mixed model ANOVA analyzing the effects of hα-syn, nicotine across the 40 min session on distance traveled: main effect of hα-syn, F_(1,64)_ = 9.380, *p* < 0.01; transitions: hα-syn x nicotine interaction, F_(1,63)_ = 5.287, *p* < 0.05, main effect of hα-syn: F_(1,63)_ = 5.841, *p* < 0.05; entries to center: hα-syn x nicotine interaction, F_(1,66)_ = 4.842, *p* < 0.05. Every measure shows a main effect of time, *p* < 0.0001. (**D**) Two-way ANOVA performed on the 20-to-40 min interval, distance traveled: main effect of hα-syn, F_(1,65)_ = 6.930, *p* < 0.05, Bonferroni’s Multiple Comparisons: hα-syn-/control vs hα-syn+/control, *p* < 0.05; transitions: hα-syn x nicotine interaction, F_(1,63)_ = 5.121, *p* < 0.05, main effect of hα-syn, F_(1,63)_ = 6.593, *p* < 0.05, Bonferroni’s Multiple Comparisons: hα-syn-/control vs hα-syn+/control *p* < 0.01; entries to center: hα-syn x nicotine interaction, F_(1,64)_ = 7.739, *p* < 0.01, Bonferroni’s Multiple Comparisons: hα-syn-/control vs. hα-syn+/control *p* < 0.01. Graphs show all data points with medians and interquartile range. * *p* < 0.05, ** *p* < 0.01, N.S., not significant. The number of animals (males and females) for each group is: hα-syn-/control (N = 18), hα-syn-/nicotine (N = 14), hα-syn+/control (N = 17), and hα-syn+/nicotine (N = 22). (**E**) Stereological quantification revealed that chronic nicotine exposure increased the number of TH+ neurons in hα-syn- but not hα-syn+ mice (t_(18)_ = 2.18, *p* < 0.05). Graph shows mean ± SEM: * *p* < 0.05. (**F**) Chronic nicotine exposure increased the number of Nurr1+ cells in the SNr of hα-syn+ mice (t_(18)_ = 2.91, *p* < 0.01). Graph shows mean ± SEM: ** *p* < 0.01. The number of animals is annotated in parentheses for each condition.

We used an inducible *Pitx3-IRES2-tTA/tetO-A53T* double transgenic mouse line, which expresses A53T human α-synuclein (hα-syn) in SN DAergic neurons [42], to investigate whether nicotine exposure improves any behavioral deficit. Breeders were given doxycycline (DOX)-containing (200 mg/kg) food pellets, in place of a regular diet, to suppress transgene expression from early embryonic stages through weaning (P21). After weaning (P21), hα-syn+ (positive) mice were placed on normal chow for 90 days (P111) to achieve optimal hα-syn overexpression exclusively in TH+ neurons (Figure 1A, arrowheads) at P120 (Figure 1B). At P120, experimental mice began a nicotine consumption (50 mg/L nicotine/1% saccharin solution) protocol for 14 days, while control animals were given 1% saccharin solution. All mice (P125) underwent BPM testing to assess locomotor function. The spatial patterns of locomotion displayed by nicotine-untreated (control) hα-syn+ mice revealed significant differences compared to hα-syn- (negative) mice (Figure 2), confirming locomotor deficits previously described in this PD mouse model (Lin et al., 2012). The hα-syn+ behavioral deficiencies included a number of locomotor and exploratory parameters (Figure 2A,B), such as distance traveled (two-way ANOVA, hα-syn main effect: F_(1,30)_ = 8.463, *p* < 0.01), transitions, the number of times mice enter one of nine regions of the testing chamber (two-way ANOVA, hα-syn main effect: F_(1,31)_ = 8.143, *p* < 0.01), and entries to center (two-way ANOVA, time x hα-syn interaction: F_(3,91)_ = 3.266, *p* < 0.05, hα-syn main effect: F_(1,32)_ = 6.114, *p* < 0.05). Remarkably, nicotine-treated hα-syn+ mice did not display these deficits when compared to nicotine-untreated hα-syn+ mice (Figure 2C, mixed model ANOVA, distance traveled: hα-syn main effect, F_(1,64)_ = 9.380, *p* < 0.01; transitions: hα-syn x nicotine interaction, F_(1,63)_ = 5.287, *p* < 0.05, hα-syn main effect, F_(1,63)_ = 5.841, *p* < 0.05; entries to center: hα-syn x nicotine interaction, F_(1,66)_ = 4.842, *p* < 0.05). Since the behavioral phenotype in the hα-syn+ mice was stronger in the latter half of the locomotor session, we analyzed minutes 20–40 separately (Figure 2D, two-way ANOVA, distance traveled: hα-syn main effect, F_(1,65)_ = 6.930, *p* < 0.05; transitions: hα-syn x nicotine interaction, F_(1,63)_ = 5.121, *p* < 0.05, hα-syn main effect, F_(1,63)_ = 6.593, *p* < 0.05; entries to center: hα-syn x nicotine interaction, F_(1,64)_ = 7.739, *p* < 0.01). The results indicate that chronic nicotine exposure attenuated hα-syn-induced locomotor deficits.

### 3.2. Chronic Nicotine Exposure Induces De Novo TH Expression in Non-DAergic Cells 

**Figure 3:**
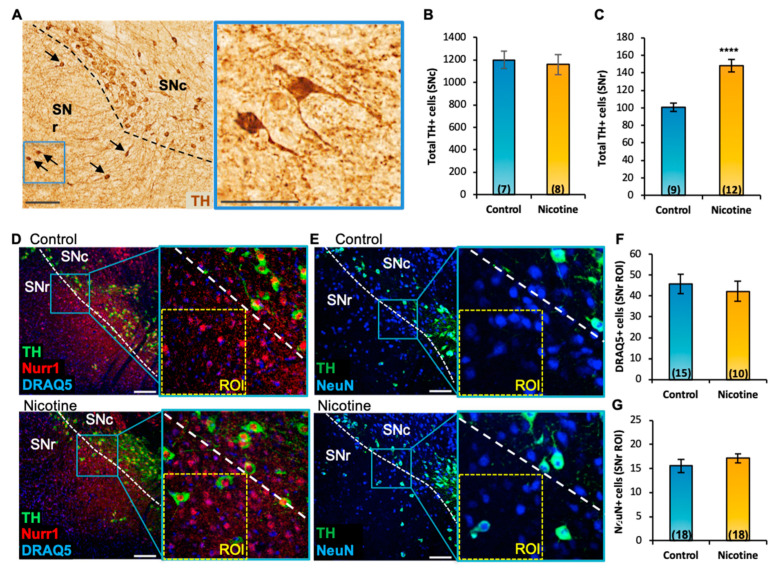
Chronic nicotine exposure increases the number of TH+ cells in the SNr without affecting the number of DRAQ5+ and NeuN+ cells. (**A**) Representative SN section of wild-type mice displaying DAB immunoreactivity for TH after chronic nicotine exposure. Arrows indicate TH+ neurons in the SNr. Scale bars = 150 μm; inset, 75 μm. (**B**,**C**) DAB stereological quantification showed that chronic nicotine exposure did not change the number of TH+ cells in the SNc (**B**) but increased the number of TH+ cells in the SNr ((**C**), t_(19)_ = 5.01, *p* < 0.0001). Graphs show mean ± SEM: **** *p* < 0.0001. (**D**,**E**) Confocal images showing TH, Nurr1, DRAQ5 (**D**), and NeuN (**E**) immunofluorescence in the SN of control and nicotine-exposed mice. (**F**,**G**) Quantification (SNr ROI) of IHC preparations shown in (**D**,**E**) revealed no change in the numbers of DRAQ5+ (**F**) and NeuN+ (**G**) cells. Graph shows mean ± SEM. The number of animals is annotated in parentheses for each condition. Scale bars = 150 μm. ROI = 150 μm × 150 μm. ROI, Region of Interest; SNc and SNr, substantia nigra compacta and reticulata.

Because nicotine directly activates SN DAergic neurons via presynaptic nAChRs [43] and exerts neuroprotective effects against PD nigrostriatal damage of DA neurons in rodents [19], we investigated the effect of two-week nicotine exposure on the number of SN neurons expressing the DA-synthesizing enzyme, tyrosine hydroxylase (TH). As previously found in wild-type mice [44], unbiased stereological quantification of TH+ neurons in hα-syn- mice revealed that chronic nicotine exposure increased the number of TH+ neurons in the SNr (Figure 2E; mean ± SEM: control = 213 ± 13, nicotine = 261 ± 15, t_(18)_ = 2.18, *p* < 0.05) but not in the SNc (Figure 3B; mean ± SEM: control = 1235 ± 58, nicotine = 1185 ± 63) compared to hα-syn+ (Figure 2E, mean ± SEM: control = 203 ± 12, nicotine = 220 ± 9). While SNr TH expression remained unchanged, nicotine-treated hα-syn+ mice showed a higher number of SNr Nurr1+ cells than control hα-syn+ mice (Figure 2F, mean ± SEM: control = 59 ± 4, nicotine = 79 ± 6, t_(18)_ = 2.91, *p* < 0.01), indicating that a reserve pool [45] of TH-negative neurons in the SNr acquired the DAergic marker, Nurr1, in response to nicotine exposure. 

To determine whether the nicotine-mediated increase in the number of TH-expressing neurons occurs through recruitment of pre-existing SNr neurons to such a DAergic phenotype, we tested the effects of 2-week nicotine exposure on the SN of adult wildtype mice (P60). After nicotine exposure, brain tissue was labelled with the DAergic TH, neuronal NeuN, and nuclear DRAQ5 IHC markers. As observed in *Pitx3-IRES2-tTA/tetO-A53T* transgenic mice (Figure 2E), stereological quantification indicated that chronic nicotine exposure in wild-type mice significantly increased the number of TH+ cells (Figure 3A, arrows, inset) in the SNr (mean ± SEM: control = 101 ± 5, nicotine = 148 ± 7, t_(19)_ = 5.01, *p* < 0.0001, Figure 2C), but not in the SNc (Figure 3B). All SNr TH-expressing cells in nicotine-treated mice expressed Nurr1 (Figure 3D, inset) and NeuN (Figure 3E, inset) markers. The increased number of TH+ neurons was not due to an increase of neuroproliferation or cell migration, as no change in the total number of DRAQ5+ (mean ± SEM: control = 46 ± 5, nicotine = 42 ± 5) and NeuN+ cells (mean ± SEM: control = 16 ± 1, nicotine = 17 ± 1) was observed in the nicotine-exposed group (Figure 3F,G). 

### 3.3. Nicotine-Induced Neurotransmitter Plasticity Occurs via Translational Induction of Nurr1 and Transcriptional TH Regulation in Non-DAergic Neurons

**Figure 4:**
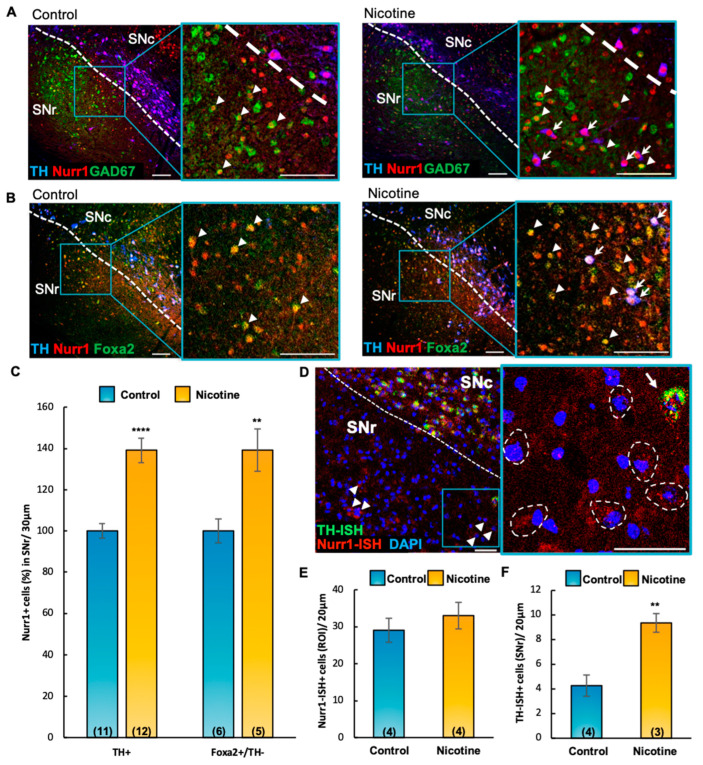
Chronic nicotine exposure increases Nurr1 expression via translational upregulation and induces *de novo* transcription of TH in non-DAergic cells. (**A**,**B**) Confocal images of coronal sections (30 μm) through the SN of control (**left** panels) and nicotine-exposed (**right** panels) mice, labeled with TH, Nurr1, GAD67-GFP, and Foxa2 markers. Insets display immunoreactive TH-/Nurr1+/GAD67+ ((**A**), arrowheads), TH-/Nurr1+/Foxa2+ ((**B**), arrowheads) cells in the SNr. Scale bars = 100 μm. (**C**) Quantification (%) of IHC preparations shown in (**A**,**B**) indicated that chronic nicotine exposure in adult (P60) mice increased the number of TH+/Nurr1+ (t_(21)_ = 5.51, *p* < 0.0001) and TH-/Foxa2+/Nurr1+ (t_(9)_ = 3.50, *p* < 0.01) cells in the SNr (arrows in **A,B**). Graph shows mean ± SEM: ** *p* < 0.01, **** *p* < 0.0001. (**D**) Confocal image of Nurr1/TH in situ hybridization (ISH) of a representative coronal section (20 μm) through the SN of nicotine-exposed adult (P150) mice. DAPI was used to label nuclei. Non-DAergic (TH-ISH negative) Nurr1-ISH+ cells (arrowheads, dashed contours in inset) and TH-ISH+/Nurr1-ISH+ cell (arrow) are observed in the SNr. Scale bars = 50 μm. (**E**,**F**) Quantification of ISH preparations shown in D (ROI, 200 μm × 200 μm) revealed no difference in the number of Nurr1-ISH+ cells between control and nicotine-exposed groups (**E**). Chronic nicotine exposure increased the number of TH-ISH+ cells ((**F**), t_(5)_ = 4.06, *p* < 0.01). Graphs show mean ± SEM: ** *p* < 0.01. The number of animals is annotated in parentheses for each condition. ROI, Region of Interest; SNc and SNr, substantia nigra compacta and reticulata. Graphs show mean ± SEM: ** *p* < 0.01. The number of animals is annotated in parentheses for each condition.

To understand the regulatory mechanism through which DAergic phenotype is acquired by non-DAergic SNr neurons in response to chronic nicotine exposure, we first evaluated the protein expression of TH, the glutamate-decarboxylase-67 (GAD67) labeling GABAergic cells, and the transcription factor Nurr1 across conditions (Figure 4A,B). Nurr1 protein, which is essential for the acquisition and maintenance of the DAergic phenotype [31,32], was detected in SNc TH+ neurons as expected. However, it was also clearly expressed in TH-negative GABAergic SNr cells, as shown by the GAD67+/Nurr1+ colocalization (Figure 4A, arrowheads). Co-activator Foxa2, which interacts with Nurr1 to promote the survival of midbrain DAergic neurons against toxic insults (Oh et al., 2015), displayed overlapping immunoreactivity in Nurr1+/TH-negative cells (Figure 4B, arrowheads). Nicotine-exposed mice (P60) displayed a significant increase in the number of Nurr1+/TH+ cells (Figure 4C, mean ± SEM: control = 100 ± 4, nicotine = 139 ± 6, t_(21)_ = 5.51, *p* < 0.0001,) and a concomitant surge in the number of TH-negative neurons co-expressing Nurr1/Foxa2, when compared to controls (Figure 4C, mean ± SEM: control = 100 ± 5, nicotine = 139 ± 10, t_(9)_ = 3.50, *p* < 0.01, Figure 4B, arrowheads). 

We then performed RNAscope in situ hybridization (ISH; Figure 4D) to investigate whether the increased level of Nurr1 and TH protein expression took place via transcriptional or translational regulation. We found no difference in the total number of Nurr1-ISH+ cells across groups (Figure 4D arrowheads and Figure 4E, mean ± SEM: control = 29 ± 3, nicotine = 33 ± 4) indicating that the increased number of Nurr1+ neurons observed in response to chronic nicotine exposure resulted from translational upregulation. In contrast, the increased number of TH-ISH+ cells in nicotine-exposed mice (Figure 4F, mean ± SEM: control = 4.25 ± 0.85, nicotine = 9.33 ± 0.88, t_(5)_ = 4.06, *p* < 0.01) reveals de novo transcription of TH mRNA in pre-existing SNr non-DAergic neurons.

### 3.4. The Pool of Neurons Recruitable for Nicotine-Induced TH Plasticity Is GABAergic 

**Figure 5:**
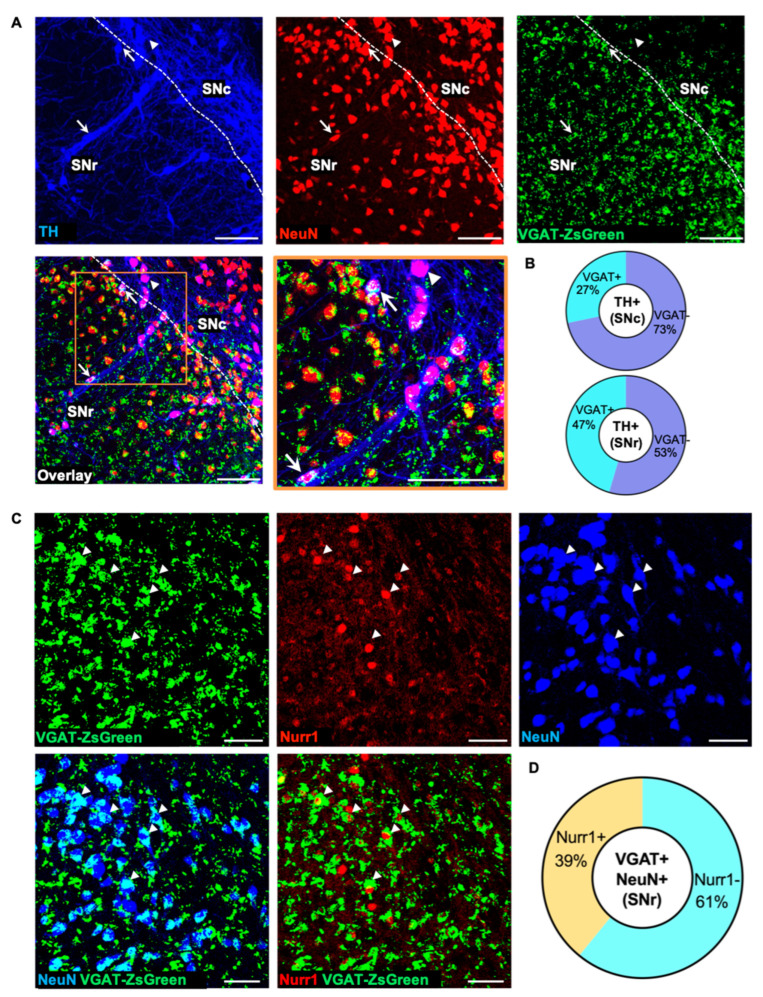
GABAergic neurons expressing Nurr1 in the SNr revealed a reserve pool recruitable to acquire a DAergic phenotype. (**A**) Representative confocal images of the SN of adult (P60) vesicular GABA transporter (VGAT)-ZsGreen mice display the distribution of TH+/VGAT+ (arrow) and TH+/VGAT- (arrowhead) neurons. Scale bars = 100 μm. (**B**) Quantification of IHC preparations shown in A indicate that 26.7 ± 3.9 % of SNc TH+ neurons and 46.8 ± 4.3% of SNr TH+ neurons express VGAT. (**C**) Confocal images of representative SNr sections show VGAT+/Nurr1+/NeuN+ colocalization (arrowheads). Scale bars = 100 μm. (**D**) Quantification of IHC preparations shown in C indicate that 38.8 ± 2.2 % of SNr VGAT+ neurons express Nurr1.

Because the SNr is primarily composed of GABAergic cells [46], we utilized the vesicular GABA transporter (VGAT)-ZsGreen transgenic mice, which constitutively expresses the ZsGreen-fluorescent protein in all GABAergic cells, to determine the fraction of neurons expressing TH and Nurr1 in the control condition. Quantification of TH/VGAT colocalization revealed a coexpression of 27 ± 4% (mean ± SEM) in the SNc and 47 ± 4% (mean ± SEM) in the SNr (Figure 5A, inset arrows; Figure 5B) in control conditions. To identify SNr neurons that are recruitable by nicotine exposure to a TH phenotype, we investigated the pool of VGAT-expressing neurons that co-localized with Nurr1 and NeuN (Figure 5C). We found that the fraction of Nurr1+/VGAT+/NeuN+ neurons (Figure 5C, arrowheads) represents 39 ± 2% (mean ± SEM) of all SNr VGAT+ neurons (Figure 5D). This pool of SNr GABAergic neurons, which display the molecular marker Nurr1 even before nicotine exposure, could represent a readily available reserve pool for nicotine-induced TH acquisition and for targeted activity-dependent manipulations.

### 3.5. A Fraction of SNr GABAergic Nurr1+ Neurons Project to the Striatum

**Figure 6:**
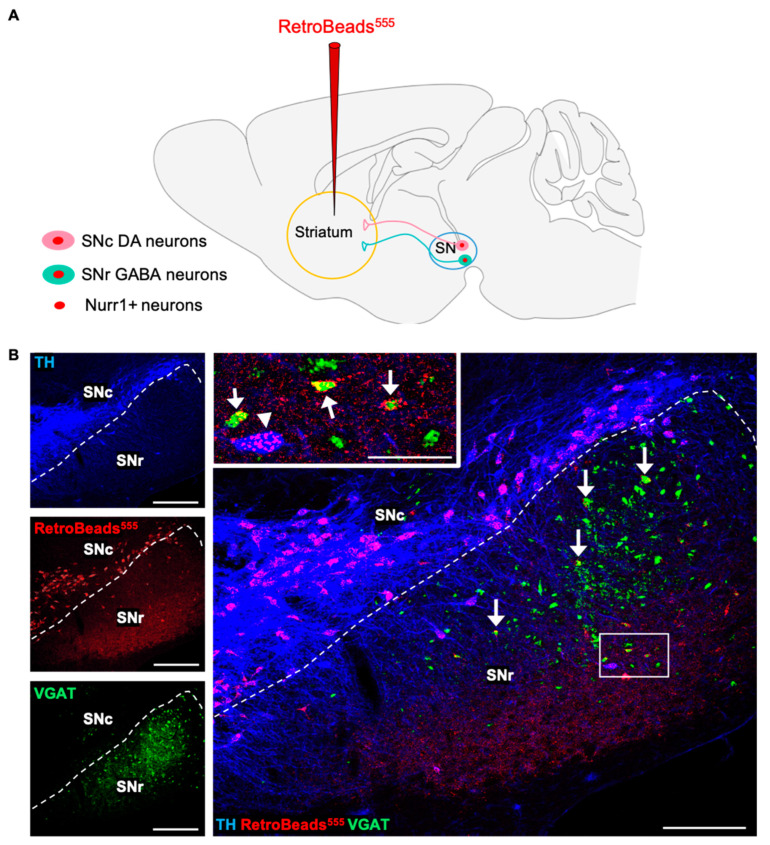
Retrograde tracing revealed the pool of SNr GABAergic neurons projecting to the striatum. (**A**) Schematic diagram illustrating retrograde tracing with RetroBeads (555 nm) injected in the striatum and transported from the neuronal terminals in the striatum back to their somata in the SN. (**B**) Confocal images of representative coronal sections through the SN of adult (P60) VGAT-ZsGreen mice. Inset (white box) shows RetroBeads detected in the somata of both TH+ (arrowhead) and VGAT+ (arrows) neurons in the SNr, revealing the connectivity of GABAergic SNr-to-striatum projection neurons. Scale bars = 200 μm, 50 μm (inset).

The nigrostriatal pathway, which is affected by neurodegeneration in PD, comprises DAergic neurons originating from the SNc and projecting to neurons located in striatum subnuclei. However, an additional fraction of nigrostriatal projections originates from GABAergic neurons located in the SNr [47,48,49]. To confirm the connectivity of GABAergic SNr-to-striatum projecting neurons, fluorescent RetroBeads (555 nm) were injected into the dorsal striatum (Figure 6A) for a retrograde tracing of striatal neuronal terminals to their SN somata. RetroBead-labelled cell bodies localized in the SNr identified SNr-to-striatum projecting neurons (Figure 6B). RetroBead accumulation was detected in both VGAT+/TH- (Figure 5B, arrows) and TH+ SNr somata (Figure 6B inset, arrowhead), in addition to all TH+ SNc-to-striatum projecting neurons. The fraction of Nurr1+ GABAergic neurons projecting to the striatum could serve as a reserve neuronal pool with the potential to acquire the TH phenotype and in turn replenish DA function in PD.

New IHC evidence showed that DAergic neurons in the SNc display a rich dendritic arborization extending into the SNr [44]. Such anatomical connectivity is in agreement with previous studies demonstrating that SNr GABAergic neurons can be electrically excited by direct activation of D_1_ and D_5_ receptors mediated by DA release from SNc DAergic dendrites [50] and nicotine-mediated activation of nAChRs [26,28].

### 3.6. Nurr1 Upregulation Is Sufficient to Ameliorate PD-Related Locomotor Deficits and Decrease the Number of hα-syn+ Neurons in the SN

**Figure 7:**
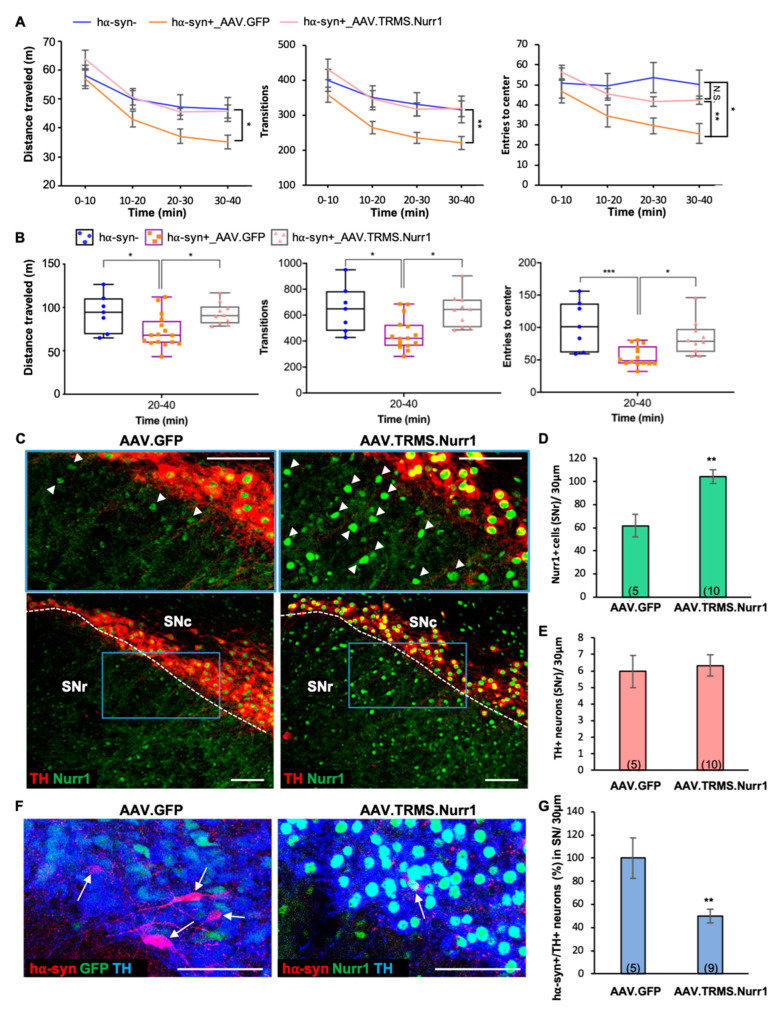
Nurr1 overexpression is sufficient to ameliorate PD-related locomotor deficits and decreases the number of hα-syn+ neurons in the SNc. (**A**) Locomotor activity (BPM) measured from 0 to 40 min of the testing session showed that pan-neuronal Nurr1-overexpression ameliorated PD-related locomotor deficits exhibited by hα-syn+_AAV.GFP mice (mixed model ANOVA for distance traveled: time x group interaction, F_(6,85)_ = 2.258, *p* < 0.05, main effect of group, F_(2,30)_ = 3.848, *p* < 0.05, Bonferroni’s Multiple Comparisons: hα-syn+_AAV.GFP vs. hα-syn+_AAV.Nurr1 *p* < 0.05 at 20–30 min and 30–40 min; transitions: main effect of group: F_(2,29)_ = 5.209, *p* < 0.05, Bonferroni’s Multiple Comparisons: hα-syn+_AAV.GFP vs hα-syn+_AAV.Nurr1 *p* < 0.05 at 10–20, 20–30 min, and *p* < 0.01 at 30–40 min; entries to center: time x group interaction, F_(6,88)_ = 4.176, *p* < 0.001, Bonferroni’s Multiple Comparisons: hα-syn+_AAV.GFP vs hα-syn- *p* < 0.05, vs hα-syn+_AAV.Nurr1 *p* < 0.01 at 30–40 min). Every measure shows a main effect of time, *p* < 0.0001. Graphs show mean ± SEM: * *p* < 0.05, ** *p* < 0.01, N.S., not significant. The number of animals for each group is: hα-syn- (N = 7), hα-syn+_AAV.GFP (N = 17), hα-syn+_AAV.Nurr1 (N = 10). (**B**) Locomotor measures (distance traveled, transitions, and entries to center) plotted for 20-to-40 min interval of the BPM testing session shown in **A** revealed a significant AAV.Nurr1-mediated rescue of the behavioral deficits displayed by hα-syn+_AAV.GFP mice. One-way ANOVA for distance traveled: F_(2,30)_ = 5.445, *p* < 0.01, Bonferroni’s Multiple Comparisons: hα-syn+_AAV.GFP vs hα-syn- *p* < 0.05, vs hα-syn+_AAV.Nurr1 *p* < 0.05; transitions: F_(2,29)_ = 6.833, *p* < 0.01, Bonferroni’s Multiple Comparisons: hα-syn+_AAV.GFP vs hα-syn- *p* < 0.05, vs hα-syn+_AAV.Nurr1 *p* < 0.05; entries to center: F_(2,28)_ = 9.293, *p* < 0.001, Bonferroni’s Multiple Comparisons: hα-syn+_AAV.GFP vs hα-syn- *p* < 0.001, vs hα-syn+_AAV.Nurr1 *p* < 0.05). Graphs show all data points with medians and interquartile range. * *p* < 0.05, *** *p* < 0.001. (**C**) Confocal images of representative SN sections indicating enhanced Nurr1-immunoreactive cell bodies (arrowheads) shown at higher magnification (insets, blue box) in mice injected with pan-neuronal Nurr1 viral vector (AAV5.TRMS.Nurr1) compared to mice injected with AAV.GFP (AAV5.TRMS.GFP). Scale bars = 100 μm. (**D**,**E**) Quantification of IHC preparations in (**C**) showed that AAV.TRMS.Nurr1 injection increased the number of Nurr1+ cells in the SNr ((**D**), t_(13)_ = 3.86, *p* < 0.01) but was not sufficient to lead to an increase in TH expression in the SNr (**E**). Graphs show mean ± SEM: ** *p* < 0.01. The number of animals is annotated in parentheses for each condition. (**F**) Confocal images of representative SN sections showing hα-syn+/TH+ neurons (arrows) in AAV.GFP- and AAV.TRMS.Nurr1-injected mice. Scale bars = 50 μm. (**G**) Quantification (%) of IHC preparations shown in (**F**) indicates that AAV.TRMS.Nurr1 injection decreased the number of hα-syn+/TH+ neurons in the SN (t_(12)_ = 3.33, *p* < 0.01). Graphs show mean ± SEM: ** *p* < 0.01. The number of animals is annotated in parentheses for each condition.

Because chronic nicotine exposure attenuated PD-related locomotor deficits and induced an increase in the expression of Nurr1 in the SNr (Figure 2), we next investigated the effects of expanding the pool of Nurr1-expressing neurons in the SN on locomotor performance in PD mice. To this aim, hα-syn+ mice were injected with AAV.TRMS.Nurr1 (hα-syn+_AAV.TRMS.Nurr1) to upregulate Nurr1 when a robust accumulation of hα-syn in TH+ neurons was already present (P111), while control hα-syn-negative mice (hα-syn-) and hα-syn+ mice were injected with a viral vector expressing GFP (hα-syn+_AAV.GFP). In agreement with our previous findings (Figure 2), hα-syn+ mice injected with AAV.GFP showed motor deficits when compared to hα-syn- mice (Figure 7A,B). A mixed-model ANOVA showed significant differences (Figure 7A) for distance traveled (time x group interaction: F_(6,85)_ = 2.258, *p* < 0.05, group main effect: F_(2,30)_ = 3.848, *p* < 0.05), transitions (group main effect: F_(2,29)_ = 5.209, *p* < 0.05), and entries to center (time x group interaction: F_(6,88)_ = 4.176, *p* < 0.001). Strikingly, hα-syn+ mice overexpressing Nurr1 did not show locomotor deficits, exhibiting a level of behavioral performance similar to hα-syn- mice. In the latter half (20–40 min) of the testing session, locomotor differences (Figure 7B, one-way ANOVA) were observed in distance traveled (F_(2,30)_ = 5.445, *p* < 0.01), transitions (F_(2,29)_ = 6.833, *p* < 0.01), and entries to center (F_(2,28)_ = 9.293, *p* < 0.001) in Nurr1-treated animals as compared to the GFP control in hα-syn+ mice. 

After behavioral testing, brain tissue was processed by IHC to investigate the effects of Nurr1 overexpression on hα-syn, TH, and Nurr1 in the SN. We confirmed that mice injected with AAV.TRMS.Nurr1 displayed enhanced Nurr1 immunoreactivity (Figure 7C, arrowheads), exemplified as an increased number of Nurr1+ cells in SNr (Figure 7D, mean ± SEM: AAV.GFP = 62 ± 10, AAV.TRMS.Nurr1 = 104 ± 6, t_(13)_ = 3.86, *p* < 0.01). While the number of TH+ neurons remained unchanged (Figure 7E, mean ± SEM: AAV.GFP = 6.0 ± 1.0, AAV.TRMS.Nurr1 = 6.3 ± 0.7), the number of hα-syn+/TH+ neurons in the SN (Figure 6F, arrows) of hα-syn+ mice injected with AAV.TRMS.Nurr1 was 50% lower than in the AAV.GFP-injected ones (Figure 7G, mean ± SEM: AAV.GFP = 100 ± 18 %, AAV.TRMS.Nurr1 = 50 ± 6 %, t_(12)_ = 3.33, *p* < 0.01), suggesting that Nurr1 overexpression resulted in a neuroprotective effect against hα-syn toxicity. 

### 3.7. Selective Nurr1 Upregulation in GABAergic Cells Is Not Sufficient to Induce a TH Phenotype

**Figure 8:**
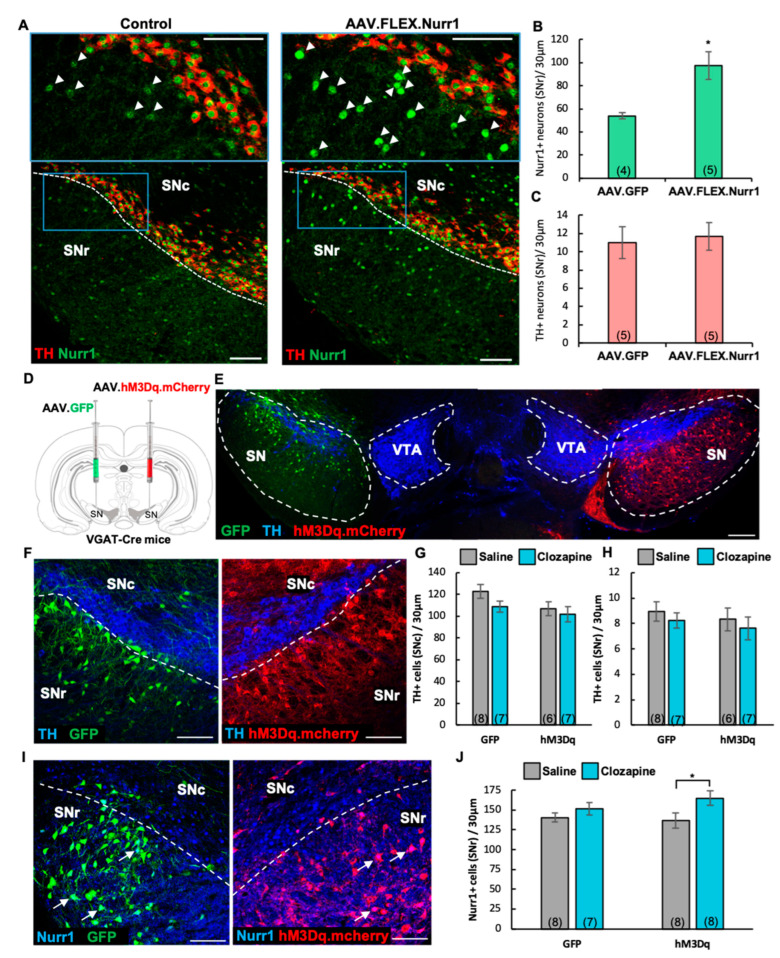
Selective Nurr1 overexpression does not elicit a TH phenotype while chronic activation of GABAergic cells is sufficient to induce Nurr1. (**A**) Confocal images of representative SN sections indicating enhanced Nurr1 immunoreactivity (arrowheads) shown at higher magnification (insets, blue box) on the side of the brain injected with Cre-dependent AAV Nurr1 miRNA (AAV.FLEX.Nurr1), when compared to the contralateral uninjected side (control). Scale bars = 200 μm. (**B**,**C**) Quantification of IHC preparations shown in A indicates that AAV.FLEX.Nurr1 injection increased the number of Nurr1+ cells in the SNr ((**B**), t_(7)_ = 2.96, *p* < 0.05) but was not sufficient to lead to an increase in TH expression in the SNr (**C**). Graphs show mean ± SEM: * *p* < 0.05. The number of animals is annotated in parentheses for each condition. (**D**) Schematic representation of the viral strategy adopted to chemogenetically activate VGAT+ neurons of the SN of VGAT-Cre mice by unilaterally injecting a Cre-dependent DREADD (hM3Dq).mCherry AAV and transfecting the contralateral side with a Cre-dependent AAV.GFP reporter (control). (**E**) Composite image of a representative section (30 μm) through the SN and VTA showing selective expression of Cre-dependent mCherry-tagged excitatory DREADD (hM3Dq) virus within the SN GABAergic neurons on the right side and Cre-dependent GFP virus transfected on the left side of a VGAT-Cre mouse brain, along with TH immunofluorescent staining. Scale bars = 200 μm. (**F**,**I**) Confocal images of representative SN sections showing TH (**F**) and Nurr1 (**I**, arrows) immunoreactivity along with GFP and hM3Dq.mCherry labeling. Scale bars = 100 μm. (**G**,**H**) Quantification of TH+ neurons of IHC preparations shown in (**F**) (SNc, two-way ANOVA, main effect of hM3Dq, F_(1,43)_ = 4.49, *p* < 0.05; SNr, unaffected by chemogenetic activation of VGAT+ neurons). Graphs show mean ± SEM. The number of animals is annotated in parentheses for each condition. (**J**) Quantification of IHC preparations shown in (**I**) revealed that DREADD (hM3Dq)-mediated activation of GABAergic cells induced an increase in Nurr1 expression (two-way ANOVA, main effect of clozapine, F_(1,14)_ = 5.325, *p* < 0.05, Bonferroni’s Multiple Comparisons: hM3Dq/saline vs hM3Dq/clozapine, *p* < 0.05). Graph shows mean ± SEM: * *p* < 0.05. The number of animals is annotated in parentheses for each condition.

Because the fraction of SNr GABAergic neurons projecting to the striatum represents a reserve pool that can acquire Nurr1 and TH phenotypes in response to nicotine-mediated activation, we tested whether Nurr1 upregulation alone, exclusively targeted to SN GABAergic cells, could induce TH plasticity. We unilaterally injected a Cre-dependent Nurr1 viral vector (AAV.FLEX.Nurr1) into the SN of VGAT-Cre mice (P60). The contralateral side was used as a control. Nurr1 immunoreactivity in the AAV.FLEX.Nurr1-injected side, as compared to the control, showed robust vector transduction (Figure 8A, arrowheads). A quantitative analysis showed that the increase in the number of Nurr1-expressing VGAT+ cells (Figure 8B, mean ± SEM: control = 54 ± 3, AAV.FLEX.Nurr1 = 98 ± 12, t_(7)_ = 2.96, *p* < 0.05) was not paralleled by an increase in the number of TH+ neurons in the SNr (Figure 8C, mean ± SEM: control = 11 ± 1, AAV.FLEX.Nurr1 = 12 ± 1), indicating that Nurr1 upregulation alone was not sufficient to induce the acquisition of TH phenotype by SNr GABAergic neurons. 

### 3.8. Chemogenetic Activation of SN GABAergic Neurons Is Sufficient to Induce the Acquisition of Nurr1 but Not TH Phenotype

Given that chronic alteration of neuronal activity [45,51,52,53] and nicotine-exposure [44,54] can elicit TH plasticity within non-DAergic interneurons, we utilized DREADDs (designer-receptors-exclusively-activated-by-designer-drugs [55]) to test whether chronic depolarization of GABAergic neurons was sufficient to induce TH plasticity. To this end, we unilaterally injected a viral vector carrying a Cre-dependent excitatory mCherry-DREADD (hM3Dq) construct into the SN of adult VGAT-Cre mice (P60) and injected the contralateral side with a Cre-dependent AAV vector expressing GFP as a control (Figure 8D,E). One month after viral infusion (P90), we began our DREADD-activation protocol by administering 0.01 mg/kg clozapine or saline as control (i.p., twice daily) for 14 days. Quantification of TH immunoreactivity (Figure 8F) showed that chronic activation of GABAergic cells was not sufficient to induce an increase in the number of TH-expressing neurons in either SNc or SNr (Figure 8G,H). However, DREADD-mediated activation was sufficient to elicit a significant increase in the number of Nurr1+ neurons exclusively in the DREADD-injected hemisphere (Figure 8I, arrows; Figure 8J, mean ± SEM: GFP/saline = 141 ± 5, GFP/clozapine = 152 ± 8, hM3Dq/saline = 137 ± 10, hM3Dq/clozapine = 165 ± 9, two-way ANOVA, clozapine main effect: F_(1,14)_ = 5.325 *p* < 0.05, Bonferroni’s Multiple Comparisons: hM3Dq/saline vs hM3Dq/clozapine, *p* < 0.05).

### 3.9. Concomitant Chemogenetic Activation of SN GABAergic Neurons and Nurr1 Upregulation Recapitulate Nicotine-Mediated Acquisition of the TH Phenotype

**Figure 9:**
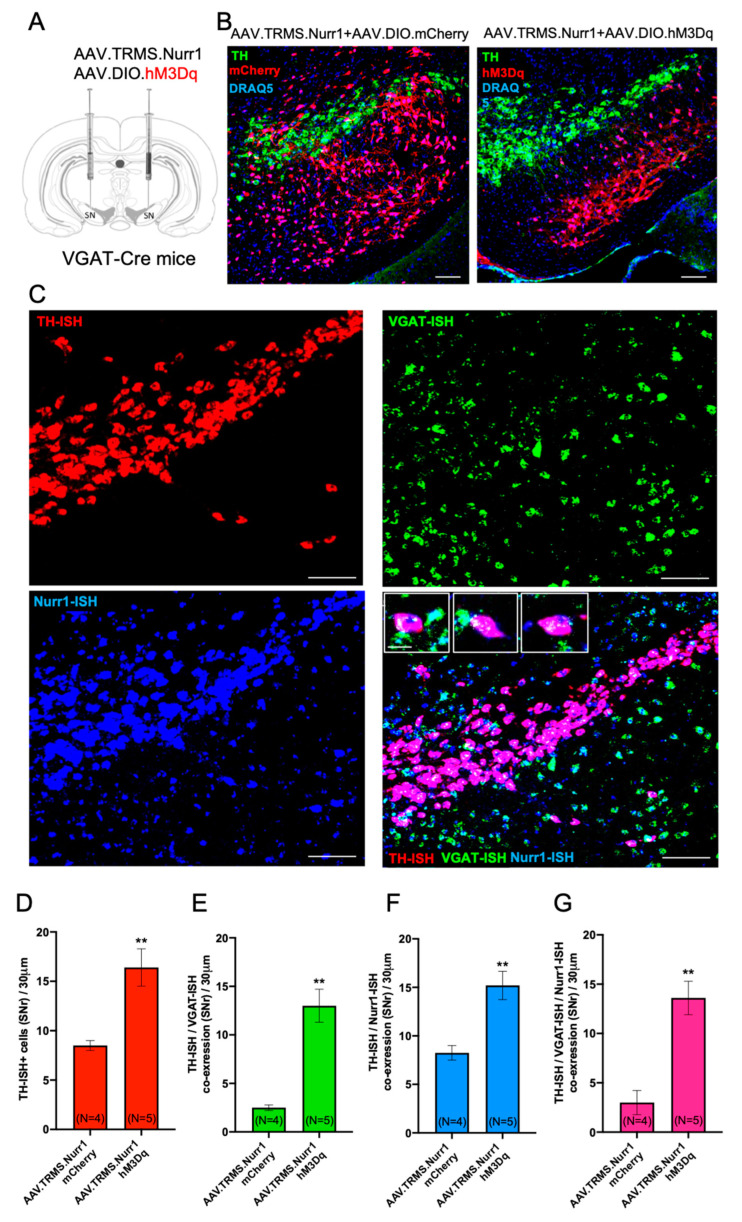
Effect of concomitant chemogenetic activation of SN GABAergic neurons and Nurr1 upregulation on TH phenotype. (**A**) Schematic representation of the viral strategy adopted to chemogenetically activate VGAT+ neurons and overexpress Nurr1 in the SN of VGAT-Cre mice by bilaterally injecting a Cre-dependent DREADD (hM3Dq).mCherry AAV and pan-neuronal Nurr1 viral vector (AAV5.TRMS.Nurr1). (**B**) Confocal images of representative SN sections (30 μm) showing selective expression of Cre-dependent mCherry (**left**) and excitatory DREADD (hM3Dq) (**right**) virus within the SN GABAergic neurons, along with TH immunofluorescent staining and nuclear staining DRAQ5. Scale bars = 100 μm. (**C**) Confocal image of TH (red), VGAT (green), Nurr1 (blue) and TH/VGAT/Nurr1 co-localization (magenta) in situ hybridization (ISH) of a representative coronal section (30 μm) through the SN of VGAT-Cre mouse injected in the SN with AAV.TRMS.Nurr1 and AAV.DIO.hM3Dq and receiving daily Clozapine i.p. injections for 2 weeks. Scale bars = 100 μm. Insets show representative SNr cells displaying TH-ISH+/VGAT-ISH+/Nurr1-ISH+/ co-expression. Scale bars = 20 μm. (**D**–**G**) Quantification of ISH preparations revealed significant differences in the number of TH-ISH+ cells (**D**), TH-ISH+/VGAT-ISH+ (**E**), TH-ISH+/Nurr1-ISH+ (**F**), and TH-ISH+/VGAT-ISH+/Nurr1-ISH+ (**G**) co-expressing neurons displayed by VGAT-Cre mice injected with either AAV5.TRMS.Nurr1 + AAV.DIO.mCherry or AAV5.TRMS.Nurr1 + AAV.DIO.hM3Dq viral vectors. Graphs show mean ± SEM: ** *p* < 0.01, Student’s *t*-test. The number of animals is annotated in parentheses for each condition.

To test whether concomitant activation of GABAergic neurons and Nurr1 overexpression is sufficient to induce TH plasticity in the SN, VGAT-Cre mice were injected with Cre-dependent excitatory mCherry-DREADD (hM3Dq) or mCherry control virus and pan-neuronal Nurr1+ (Figure 9A,B). Four weeks after viral injections, mice received 0.01 mg/kg clozapine i.p., twice daily for 14 days to induce chronic activation of GABAergic cells in the SN. RNAscope in situ hybridization showed that Nurr1-hM3Dq mice displayed a significant increase in the total number of TH-ISH cells as well as TH/VGAT-ISH co-expression in the SN (Figure 9D, mean ± SEM: Nurr1+-mCherry = 8.5 ± 0.5, Nurr1-hM3Dq = 11 ± 1.7, t_(7)_ = 3.61, *p* < 0.01p; Figure 9E Nurr1+-mCherry = 2.5 ± 0.2, Nurr1-hM3Dq = 13 ± 1.7, t_(7)_ = 5.39, *p* < 0.01p) compared to Nurr1-mCherry mice. In addition, TH/Nurr1-ISH co-expression and TH/VGAT/Nurr1+ co-expression increased in Nurr1-hM3Dq mice compared to Nurr1-mCherry mice (Figure 9F, mean ± SEM: Nurr1+-mCherry = 8.2 ± 0.7, Nurr1-hM3Dq = 15.2 ± 1.4, t_(7)_ = 3.89, *p* < 0.01p; Figure 9G Nurr1+-mCherry = 3.0 ± 1.2, Nurr1-hM3Dq = 13.6 ± 1.6, t_(7)_ = 4.82, *p* < 0.01p). 

## 4. Discussion

Our findings show that chronic nicotine exposure attenuates locomotor deficits in a human-α-syn-expressing mouse model of PD [42] and primes a GABAergic neuronal pool in the SNr to a novel form of neuroplasticity, culminating in the acquisition of the TH phenotype. Nicotine activation of nAChRs in the nigrostriatal pathway elicits an increase in calcium influx [23,24,25] and induces neuronal depolarization [21,22]. Previous studies have reported on the dense distribution of nAChRs in both DAergic and GABAergic neurons in the SN [26,28], suggesting a potential activity-dependent mechanism in the regulation of DAergic circuits in nicotine-mediated protection against PD [19,56,57,58]. Specifically, 99% of SNr GABAergic neurons express both α_4_* nAChR readily available for nicotine activation [26,28] as well as DA D_1_ and D_5_ receptors which are tonically excited by dendritically released DA from the SNc DAergic neurons, forming a relatively short SNc-to-SNr DAergic pathway [50]. Such dendro-dendritic connectivity and the specific receptor expression displayed by descending SNc DAergic dendrites into the SNr GABAergic neuropil provide the opportunity for the nigrostriatal circuit to signal common instructions to both the DAergic and the GABAergic pathways when DA function needs a physiological boost. These conditions have been shown to be a requirement for activity-dependent recruitment of non-DAergic neurons to a DAergic phenotype [45]. Chronic nicotine exposure could elicit the recruitment of SNr GABAergic neurons to Nurr1 and TH phenotypes through at least two potential activity-dependent signaling mechanisms: (a) nicotine directly activates the α_4_* nAChRs localized on the SNr GABAergic neurons; or (b) as nicotine activates SNc DAergic neurons via nAChRs, the DA released from the dendrites activates SNr GABAergic neurons through D_1_ and D_5_ receptors. Both mechanisms could, in principle, initiate the calcium-mediated reprogramming required to induce the TH phenotype in the SNr GABAergic neurons, as previously found in neurons of the SNc [59]. 

Electrical activity and calcium signaling have significant roles in regulating various forms of neuroplasticity, including priming neurons with the molecular memory of early nicotine exposure [54] and neurotransmitter reprogramming [51,60,61]. Sustained alteration in circuit activation by either experimental manipulation or natural sensory stimuli can induce neurotransmitter plasticity in the brain, affecting behavior [52,53,54,62,63,64]. While SNr GABAergic neurons undergo a significant upregulation of α_4_* nAChR subtype in response to chronic nicotine exposure, the level of these receptors in SNc DAergic neurons remains unchanged [28]. Selective upregulation of α4* nAChR levels in the SNr might bring the level of calcium transients in these neurons to a threshold sufficient to signal and initiate neurotransmitter plasticity in response to chronic nicotine exposure, providing another layer of specificity in the recruitment of GABAergic neurons of the SNr and not the SNc to nicotine-mediated TH plasticity. 

In this study, we identified a reserve pool of Nurr1-expressing GABAergic neurons in the SNr that undergoes nicotine-mediated TH respecification; a phenomenon that might represent a layer of functional protection against PD. Our findings provide an important parallel to previously reported phenotypic shift of pre-existing GABAergic neurons to express TH in adult macaques following treatment with MPTP, a neurotoxin that induces DA depletion mimicking PD [65]. Importantly, we confirmed by retrograde tracing that part of the nigrostriatal projection originates from SNr GABAergic neurons and demonstrated that these neurons share the same target as DAergic neurons in the SNc. Therefore, these SNr GABAergic neurons could serve the role as a reserve pool that could gain the DA-synthesizing enzyme and potentially rescue the DAergic loss of function caused by neurodegeneration of SNc DAergic neurons. Given that this form of TH plasticity also occurs in the SN of primates [66] in response to DAergic neuron loss, understanding the mechanism of nicotine-induced TH respecification in the SNr has tremendous translational value in the constant search for new approaches aimed at replenishing DA function in PD. 

As chronic nicotine exposure leads to improved locomotion in hα-syn+ mice and concomitantly increases the number of Nurr1+ cells in the SNr, we further investigated the effect of the induced upregulation of Nurr1 expression in the SN of hα-syn+ mice. We found that Nurr1 overexpression was sufficient to ameliorate PD-related locomotor deficiencies. This is in agreement with previous studies highlighting the role of Nurr1 in pathogenesis of PD and its potential as a therapeutic target [37,38]. Our results here show, for the first time, that Nurr1 overexpression elicits protection against PD-related locomotor dysfunctions through a reduction of the number of hα-syn-expressing TH+ neurons. In addition, the upregulation of Nurr1/Foxa2 co-expression in non-DAergic neurons observed after chronic nicotine exposure could represent part of the priming mechanism generating a molecular memory of nicotine exposure aimed at expanding the reserve pool of potential neurons equipped to undergo the TH genetic program when properly motivated by a persistent nicotine exposure. 

Given the established neurodegenerative effects of abnormal α-synuclein [67] and the therapeutic effect of Nurr1 [37,38], chronic nicotine exposure might slow down the etiology of neurodegeneration by reducing and attenuating α-syn toxicity. 

Our chemogenetic approach implemented to selectively and chronically depolarize GABAergic neurons was not sufficient to induce TH plasticity in these neurons; however, it revealed a way to experimentally induce an expansion of the reserve pool of Nurr1+ neurons in the SNr that is available for recruitment to TH phenotype acquisition. Indeed, selective Nurr1 upregulation and chronic activation of GABAergic neurons together recapitulated TH respecification observed in the SNr of mice chronically exposed to nicotine. 

Future studies will uncover all key players required for a combinatorial manipulation that would successfully induce SNr GABAergic neurons to acquire the TH phenotype even in PD mice. Since the SNr GABAergic fraction of the nigrostriatal pathway is completely spared by PD-associated neurodegeneration, the gain of the DAergic phenotype could in principle replenish DA in the striatum. Establishing effective manipulations targeted to induce TH plasticity in GABAergic neurons of the nigrostriatal pathway could represent a paradigm shift in developing a novel approach for PD treatment.

## 5. Methods and Materials

### 5.1. Mice

Mice were from a C57BL/6J genetic background, except for GAD67- GFP mice that were from a CD1 background. The Gad1-tm1.1Tama (GAD67-GFP knock-in) mouse line was provided by Y. Yanagawa (Gunma University Graduate School of Medicine, Japan). Mice were heterozygous for insertion of the gene encoding GFP under the control of the GAD67 gene promoter. They were used to label the inhibitory GABAergic neurons in the SN by enhancing the GFP signal with an anti-GFP antibody. Adult (P60) mice, obtained from the Jackson Laboratory (Bar Harbor, ME, USA) weighing 25–35 g, were used in this study. VGAT-IRES-Cre knock-in mice (STOCK Slc32a1tm2(cre)Lowl/J, Jackson stock 016962) were used for chemogenetic manipulations. To induce the expression of the ZsGreen label in GABAergic cell bodies, VGAT-Cre mice were bred with reporter mice (B6.Cg-*Gt(ROSA)26Sor^tm6(CAG−ZsGreen1)Hze^*/J, Jackson stock 007906) that express CAG-promoter-driven enhanced green fluorescent protein (ZsGreen1) following Cre-mediated recombination. The *Pitx3-IRES2-tTA/tetO-A53T* double transgenic mouse line, which expresses mutant (SNCA*A53T) human α-synuclein in midbrain dopaminergic neurons, was previously characterized [42] and generously provided by Dr. Cai at NIH. By crossing the driver line, *Pitx3-IRES-tTA* mice (B6.129(FVB)-*Pitx3^tm1.1Cai^*/J, Jackson stock 021962), with the responder line, *tetO-A53T*, which encodes a human α-synuclein mutant gene under the control of a *tetO* promoter (STOCK Tg(tetO-SNCA*A53T)E2Cai/J, Jackson stock 012442), the expression of A53T α-synuclein in the SN dopaminergic neurons was driven using a binary tetracycline-dependent “tet-off” inducible gene expression system. Breeders were given doxycycline (DOX)-containing (200 mg/kg) food pellets (Bio-Serv, Flemington, NJ, USA), in place of a regular diet, to suppress transgene expression from early embryonic stages through weaning (P21). Adult mice, weighing 20–30 g, were used to investigate either the accumulation of human α-synuclein (P30 through P180) or the effects of nicotine exposure. Mice of the responder line *tetO-A53T* were used as controls (hα-syn−). Mice were housed in accordance with the guidelines of the University of California San Diego Institutional Animal Care and Use Committee. Experiments involved male and female adult mice (9 to 16 weeks old) maintained either in 12:12 light/dark cycles (12 h light and 12 h dark) with food and water available ad libitum.

### 5.2. Stereotactic Injections

Mice were anesthetized with 3% isoflurane and placed in a stereotactic apparatus (David Kopf Instruments, Tujunga, CA, USA, Model 900HD Motorized Small Animal Stereotaxic). Brain injections were performed during a continuous flow of 1% isoflurane. For Chronic chemogenetic activation via designer receptor exclusively activated by designer drugs (DREADD) [55], adult VGAT-Cre mice (P60 or P90) underwent bilateral stereotactic injections (500 nL/side, 1.0 × 10^13^ GC/mL) into the SN (AP = −3.08 mm, L = ±1.38 mm, DV = −4.66 mm) with a control virus (AAVDJ.Syn1.DIO.eGFP, Salk Institute, La Jolla, CA, USA) on one side and the excitatory DREADD receptor-encoding virus (AAV5.hSyn.DIO.hM3Dq.mCherry, Addgene, Watertown, MA, USA) on the contralateral side. For Nurr1 overexpression pan-neuronal adult (P111) hα-syn+ mice were injected in the SN with either AAV5.TRMS.Nurr1 [68] (300 nL, 1.0 × 10^13^ GC/mL) or with AAV.TRMS.GFP (300 nL, 1.6 × 10^13^ GC/mL) control virus. For Cre-dependent Nurr1 overexpression in GABAergic neurons of the SN, adult (P90) VGAT-Cre mice were injected with AAV9.FLEX.Nurr1 (300 nL, 1.0 × 10^13^ GC/mL); the control group was injected with AAV9.FLEX.GFP (300 nL, 1.0 × 10^13^ GC/mL). To allow diffusion of the injected virus, the injection needle remained in place for 8 min before removal. After surgery, mice were injected with 0.1 mg/kg/24 h buprenorphine as analgesia. Viral incubation occurred for 4–6 weeks after surgery. 

For retrograde tracing fluorescent RetroBeads (80 nL, 555 nm, LumaFluor, Inc., Durham, NC, USA) were unilaterally injected in the striatum (AP = −0.20 mm, L = ±2.60 mm, DV = −3.00 mm) of VGAT-ZsGreen mice. Mice were sacrificed after 10 days of recovery to allow adequate time for retrograde transport of RetroBeads from the striatal terminals to the soma of SN neurons. 

### 5.3. Drug Treatment and Blood Collection

For chronic nicotine exposure two groups of adult GAD67-GFP (P60), VGAT-Cre (P60), or hα-syn+ (P111) mice underwent chronic nicotine exposure for two weeks. Drinking water was replaced with a solution of 50 mg/L nicotine in 1% saccharin (nicotine-treated group) or with 1% saccharin solution (control condition). Animals were sacrificed after the two-week treatment. The amount of fluid intake was measured daily throughout the experiment; the initial and final body weight of the mice were also measured. Plasma nicotine metabolite levels (18 ng/mL) were assayed by HPLC (NMS Labs, Horsham, PA, USA). Blood samples were collected from adult mice after two weeks of nicotine treatment from mice anesthetized with ketamine/xylazine and assayed for plasma nicotine metabolite, cotinine, titer (18 ng/mL) by high performance liquid chromatography (NMS Labs).

For chronic chemogenetic activation via DREADD, Clozapine (0.01 mg/kg; MP Biomedicals, Santa Ana, CA, USA) was dissolved in 0.1% dimethyl sulfoxide (DMSO) in sterile saline, or vehicle (sterile saline) was administered intraperitoneally (IP) to VGAT-Cre mice twice daily for 14 days.

### 5.4. Immunohistochemistry and In Situ Hybridization

For immunohistochemistry (IHC), mice were anesthetized with a ketamine/xylazine cocktail (10 mg/kg) delivered via i.p. injection. Animals were transcardially perfused with room-temperature phosphate buffered saline (1X PBS) followed by ice-cold 4% paraformaldehyde (PFA). Brains were incubated with 4% PFA overnight at 4 °C and transferred to 30% sucrose for 48–72 h until sunk. Brains were then serially sectioned at 30–20 μm using a Leica microtome (SM 2010R) and collected in PBS. 

Per animal, 6–8 brain sections encompassing SN were incubated with antibodies (listed in Table 1) in blocking solution (1X PBS containing 5% normal horse serum and 0.3% Triton X-100) for 24 h at 4 °C. Sections were then washed 3 × 10′ in PBS, then incubated in secondary antibodies in blocking solution for 1 h at room temperature, washed 3 × 10′ in PBS, mounted on a positively charged Superfrost Plus glass slide (Fisherbrand, Pittsburgh, PA, USA) with 0.2% gelatin in PBS, cover-slipped with mounting medium (Fluoromount-G^®^, SouthernBiotech, Birmingham, AL, USA) with or without DRAQ5 (1 μm/mL, BioStatus, Loughborough, UK), and sealed with nail polish for permanent storage and imaging. For RNAscope in situ hybridization detection and labeling of TH, Nurr1 and VGAT mRNA transcripts were performed following manufacturer instructions (Advanced Cell Diagnostics, Newark, CA, USA). Sections were counterstained with DAPI and slides cover-slipped using Fluoromount-G mounting medium. Images were acquired at 20X magnification with a Leica TCS SPE confocal microscope (Leica Microsystems, Deerfield, IL, USA). Maximized fluorescence final images were obtained from a total of 11 Z-stacked layers 2 μm away from each other. Cells were counted by an investigator blind to treatment using the Adobe Photoshop CC counting tool.

For colorimetric DAB (3,3-Diaminobenzidine)-based IHC, free-floating sections were washed three times (10 min per wash) in PBS, then incubated in Avidin-Biotin Complex (Vector laboratories, Newark, CA, USA) solution (1X PBS containing 0.3% Triton X-100, 2% NaCl, and 1% of Reagents A and B from the vectastain ABC kit) for one hour, washed again (3 × 10 min), and incubated in fresh DAB solution (25 mg/mL) for approximately 3 min depending on the speed of the reaction. Sections were rinsed twice quickly and washed for 20 min in PBS before mounting on glass slides. Sections on slides were dried in a fume hood, then defatted in 1:1 chloroform:ethanol solution for two hours, and progressively rehydrated in 100% ethanol, 95% ethanol, and distilled water. Sections were then counterstained in 0.1% cresyl violet solution for 30 min, rinsed quickly in distilled water, dehydrated in 95% ethanol for three minutes, in 100% ethanol twice for five minutes each, and cleared (2 × 5 min) in Xylenes (brand). Slides were cover-slipped with permanent mounting medium Cytoseal^TM^ 60 (Thermo Scientific, Waltham, MA, USA). Images were acquired using Hamamatsu Nanozoomer 2.0HT Slide Scanner. The quantification was performed by unbiased stereology (using a Leica DM4 B microscope and Stereologer 2000 software, version SS-15, MBF Bioscience, Williston, VT, USA).

An unbiased count of DAB-stained neurons was performed using a Leica DM4 B microscope and Stereologer2000 software. The investigator was blind to experimental conditions. An exhaustive count of SNc TH-immunostained neurons (Slab Sampling Interval = 1, Total Number of Sections = 20, Section Sampling Interval = 2) was performed with a 63X oil objective after outlining the SNc with a 10× objective. The count was performed using a total of 100 dissectors (Frame Area: 5000 μm^2^, Frame Height: 20 μm, Guard Height: 2 μm, Frame Spacing: 100 μm). A neuron was considered as positive for immunoreactivity when its nucleus fell inside the dissector borders without touching the exclusion lines. For SNr TH-immunoreactive neurons (Figure 1E: Slab Sampling Interval = 1, Total Number of Sections = 24, Section Sampling Interval = 3; Figure 2C: Slab Sampling Interval = 1, Total Number of Sections = 20, Section Sampling Interval = 2), a rare event protocol was used to perform an exhaustive count with a 10× objective (Frame Area: 5000 μm^2^, Frame Height: 20 μm, Guard Height: 2 μm, Frame Spacing: 100 μm).

### 5.5. Behavioral Testing

The causal links between changes in DA expression and behavior have been documented for other DA networks [69]. To assess PD-related behavioral deficits associated with A53T-expression and effects of nicotine treatment, mouse behavioral pattern monitor (BPM, San Diego Instruments, San Diego, CA, USA) chambers were used to measure locomotor activity and investigatory behavior [7]. This system collects data encompassing total traveling distance, rearing movements, duration spent in the center, number of entries to the center, transitions (number of times mouse entered one of nine regions), and number of investigatory nosepokes (holepokes). A mouse BPM chamber is a clear Plexiglas box containing a 30 × 60 cm holeboard floor. Each chamber is enclosed in a ventilated outer box to protect it from outside ambient noise and light. The location of the mouse is obtained from a grid composed of a 12 × 24 X-Y array of infrared photobeams that are placed 1 cm above the floor. There are 8 square sectors (15.2 cm wide) in each chamber. Crossovers between each sector are defined as movements between any of these sectors. Each chamber is also divided into 9 regions unequal in size that are used primarily to define entries into the corners and the center. Rearing is detected by an array of 16 photobeams placed 2.5 cm above the floor. Holepokes are detected by 11 1.4 cm holes in the chamber (3 in the floor and 8 in the wall), each equipped with an infrared photobeam. The status of the photobeams is sampled every 55 ms. A change in the status triggers the storage of information in a binary data file together with the duration of the photobeam status. Subsequently, the raw data files are transformed into (x, y, t, event) ASCII data files composed of the (x, y) location of the animal in the mouse BPM chamber with a resolution of 1.25 cm, the duration of each event (t) and whether a holepoke or rearing occurred (event). ASCII data were then exported into Microsoft Excel files for subsequent statistical analyses with GraphPad Prism 8.4.0.

A total of eight chambers was used, each chamber measuring one mouse per session (40 min). The BPM test was conducted after 14 days of nicotine administration and performed over 2 days, with male mice tested on the first day and female mice on the second day to avoid disruption of behavior by scent from the opposite sex. The animals were brought into the testing room 1 h before testing. During testing, a white noise generator produced background noise at 65 dB. The chambers were cleaned thoroughly between testing sessions. 

For chronic nicotine exposure experiments, mice were divided into four groups (two genotypes: hα-syn+, hα-syn−; two treatments: control, nicotine). To test the effects of Nurr1 overexpression on behavior, hα-syn+ mice were divided into three groups (two genotypes: hα-syn+, hα-syn−; two types of SN viral injections: AAV.GFP, AAV.Nurr1).

### 5.6. Experimental Design and Statistical Analysis

Data were analyzed using two-tailed Student’s *t*-test and one-way, two-way, or mixed model analysis of variance (ANOVA), as appropriate for each experiment. A criterion based on z-score was used to detect outliers prior to running the ANOVA. The level 0.01 was chosen as the decision criterion for the z-score of 3.291 beyond which a datum is considered an outlier. Significant main effects and interactions were followed by Bonferroni’s Multiple Comparisons tests. Data are represented by mean and standard error in bar and line graphs, or by the median and interquartile range with all data points in box and whisker plots. The alpha level was set to 0.05 for all analyses. Appropriate sample size for each experiment was determined with standard Cohens’s d power analysis with target power set to 0.8 and alpha level to 0.05. Data were analyzed with IBM SPSS Statistics 26.0 and GraphPad Prism 8.4.0. Graphs were generated with Microsoft Excel and GraphPad Prism 8.4.0.

## Figures and Tables

**Table 1:** The manufacturer, the lot number (#), and the working solution used for each antibody used in this study.

Antibody	Manufacturer Lot #	Dilution Ratio
Chicken anti GFP	Invitrogen, A10262	1:500
Chicken anti GFAP	Invitrogen, AB5541	1:1000
Goat anti Foxa2	Boster, A01032	1:250
Guinea Pig anti NeuN	Millipore, AB2251	1:1500
Mouse anti TH	Millipore, MAB318	1:1000
Mouse anti α-syn	Santa Cruz, sc-12767	1:500
Rabbit anti Nurr1	Santa Cruz, sc-990	1:300
Sheep anti TH	Novus, NB300-110	1:1000

## Data Availability

Research data is stored in our laboratory storage unit.
